# Monthly administration of rituximab is useful for patients with ocular adnexal mucosa-associated lymphoid tissue lymphoma

**DOI:** 10.1038/bcj.2014.65

**Published:** 2014-09-12

**Authors:** T Mino, K Mihara, T Yoshida, Y Takihara, T Ichinohe

**Affiliations:** 1 Department of Hematology and Oncology, Research Institute for Radiation Biology and Medicine, Hiroshima University, Hiroshima, Japan; 2 Department of Stem Cell Biology, Research Institute for Radiation Biology and Medicine, Hiroshima University, Hiroshima, Japan

Ocular adnexal mucosa-associated lymphoid tissue-type lymphoma (OAL) is a distinct category of indolent B-cell non-Hodgkin's lymphoma.^1^ OAL is commonly found in clinical stage IE or IIE at diagnosis, and is associated with a favorable prognosis.^2, 3, 4, 5^ However, patients often require treatment because of cosmetic problems, discomfort or pain. Although local irradiation therapy has been frequently used as a treatment option for OAL, it is associated with high risks of complications, such as dry eye, cataract, decreased visual acuity, conjunctivitis, conjunctival ulcer, retinopathy, optic neuropathy and cancer.^2,6, 7, 8, 9, 10^ The repeated administration of rituximab, weekly four times, is an effective alternative associated with high response rates and lower risks of complications. However, a high rate of relapse has reportedly been a marked concern with this modality.^2,11,12^ Accordingly, an optimal treatment strategy with fewer adverse effects and sustainable efficacy is required to treat patients with OAL because such patients show a more favorable prognosis. Here, we retrospectively compared the efficacy and adverse effects of the monthly administration of rituximab with that of radiotherapy as the initial treatment for OAL. We showed that monthly rituximab may be more useful in view of the better event-free survival (EFS) and fewer adverse effects on treating patients with OAL, compared with radiotherapy and weekly infusion four times of rituximab.

Table 1 shows the characteristics of patients diagnosed with OAL in our department between 2008 and 2013. Informed consent for each treatment was obtained from all patients. Responses were assessed by whole-body computed tomography, FDG-positron emission tomography, magnetic resonance imaging and ophthalmologic tests after the completion of eight cycles of rituximab monotherapy. Ten patients with OAL received rituximab alone at 375 mg/m^2^ per day intravenously every 4 weeks for up to eight cycles. Alternatively, seven OAL patients received local irradiation of a single eye or both eyes as a historical control in our hospital. As shown in Table 1, the median age was 70 (36–79 years) in the group treated with rituximab alone, and 73 (43–82 years) in the group receiving irradiation. Proportions of females and CSIIE patients were not significantly different between the two groups (*P*=1.0 for females; *P*=0.33 for CSIIE by Fisher's exact test). All rituximab-treated patients (*n*=10) achieved and maintained complete remission during the clinical follow-up (6–29 months). There were no critical adverse events associated with the monthly infusion of rituximab. As all patients achieved complete remission and were alive during the follow-up period, EFS, defined as survival without evidence of disease progression or relapse, was calculated by Kaplan–Meier methods according to the treatment modalities.

EFS with the monthly administration of rituximab was sustained for a prolonged period (Figure 1). Two of eight patients relapsed in 24–26 months after the completion of radiotherapy. All patients treated with irradiation complained of dacryoadenitis, conjunctivitis, visual acuity loss or cataract. EFS of the patients receiving irradiation and monthly rituximab monotherapy is shown in Figure 1. These results demonstrated that EFS rates with the monthly administration of rituximab and that of radiotherapy were comparable (*P*-value=0.37).

The recent availability of rituximab has improved the EFS, progression-free survival (PFS) and overall survival of patients with B-cell non-Hodgkin's lymphoma. The frequency and severity of its side effects are non-significant during mono-treatment. Thus, rituximab is currently one of the prerequisite drugs for patients with B-cell non-Hodgkin's lymphoma, especially for the aged. It was reported that four weekly cycles of rituximab at doses of 375 mg/m^2^ as induction therapy were significantly effective in patients with OAL, but early recurrence was common.^2,11,12^ Ardeshna *et al.*^13^ reported that four weekly rituximab doses of 375 mg/m^2^ followed by maintenance therapy every couple of months for 2 years improved PFS compared with induction therapy alone for advanced and asymptomatic follicular lymphoma, which belongs to indolent lymphoma, in which mucosa-associated lymphoid tissue-type lymphoma is classified. Notably, it is of interest that the 2-year PFS in the rituximab induction group was equivalent to the 4-year PFS in the maintenance group, and furthermore, the 4-year PFS in the former was almost the same as the 6-year PFS in the latter,^13^ suggesting that the duration of exposure to even a low concentration of rituximab is more critical than its concentration. The alternative modality of radiotherapy is a promising tool for OAL, as the local control rate was reportedly extremely high (85–100%).^2,6, 7, 8, 9, 10,14,15^ However, the risk of distant relapse is high at 10–25% and there are frequent adverse effects, such as long-term toxicity causing cataract (30–50%), xerophthalmia (20–40%), ischemic retinopathy, optic atrophy, corneal ulceration and neovascular glaucoma, as well as immediate toxicity including conjunctivitis, conjunctival ulcer and dry eyes.^2,6, 7, 8, 9, 10,14,15^ Patients with OAL receiving local irradiation also suffer from dry eye, cataract and conjunctivitis based on our experience. As OAL is associated with quite a favorable prognosis, therapeutic options that preserve the quality-of-life of patients with less adverse effects are chosen. Thus, we decided on the monthly administration of rituximab and obtained desirable results regarding the efficacy, EFS and reduced adverse effects in patients receiving monthly rituximab treatment. Another intriguing phenomenon is that the efficacy of rituximab may vary depending on the treatment schedule. Manipulation of the increased number of effector cells, such as NK cells and monocytes expressing Fcγ receptor, which binds to the Fc of rituximab, might be useful for patients with B-cell non-Hodgkin's lymphoma cells regarding the efficacy of rituximab. Taken together, our treatment modality may provide a more beneficial treatment outcome with less adverse effect.

## Figures and Tables

**Figure 1:**
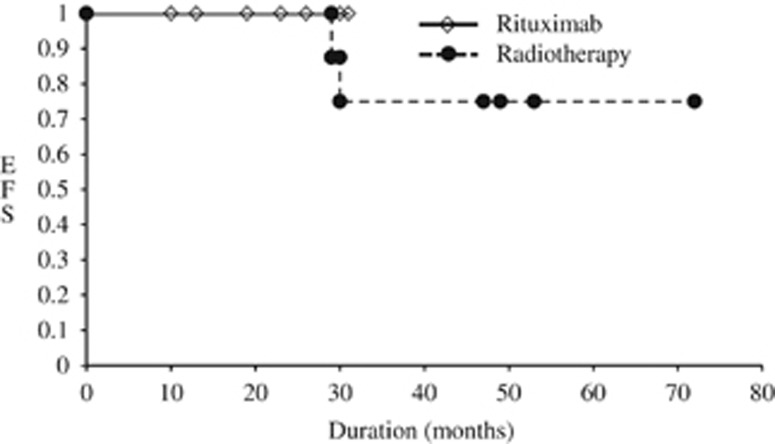
EFS with monthly administration of rituximab and radiotherapy. EFS of patients receiving irradiation and rituximab therapy is shown. Dotted and solid lines indicate EFS in patients with radiotherapy or rituximab alone, respectively.

**Table 1:** Characteristics of patients diagnosed with OAL

*Pt. no.*	*Age (years)*	*Gender*	*CS*	*Rituximab (cycles)*	*RT (Gy)*	*Outcome*	*Relapse*	*Complications (⩾grade 2)*
1	36	M	IE	8	NA	CR	ND	ND
2	70	F	IE	8	NA	CR	ND	ND
3	47	F	IIE	8	NA	CR	ND	ND
4	79	M	IE	8	NA	CR	ND	ND
5	76	M	IE	8	NA	CR	ND	ND
6	74	M	IIE	8	NA	CR	ND	ND
7	71	M	IE	8	NA	CR	ND	ND
8	46	F	IE	8	NA	CR	ND	ND
9	65	F	IE	8	NA	CR	ND	ND
10	64	M	IIE	NA	30.6	CR	ND	Conjunctivitis
11	43	F	IIE	NA	30.6	CR	ND	Cataract
12	81	M	IE	NA	30.0	CR	ND	Conjunctivitis
13	82	F	IE	NA	30.0	CR	ND	Cataract, conjunctivitis
14	77	F	IIE	NA	30.6	CR	ND	Cataract, decreased visual acuity
15	67	M	IE	NA	30.6	CR	ND	Cataract
16	81	F	IIE	NA	45.0	CR	Locally relapsed	Dacryoadenitis
17	69	M	IE	8	30.0	CR	Locally relapsed	Conjunctivitis, visual acuity

Abbreviations: CR, complete remission; CS, clinical stage; NA, not applicable; ND, not determined; RT, dose of radiotherapy.

Patient no. 17 received eight cycles of rituximab following relapse after achieving CR by RT.
